# Secretory clusterin promotes hepatocellular carcinoma progression by facilitating cancer stem cell properties via AKT/GSK-3β/β-catenin axis

**DOI:** 10.1186/s12967-020-02262-7

**Published:** 2020-02-14

**Authors:** Wenjie Zheng, Min Yao, Mengna Wu, Junling Yang, Dengfu Yao, Li Wang

**Affiliations:** 1 Research Center of Clinical Medicine, Affiliated Hospital of Nantong University, 20 Xisi Road, Nantong, 226001 Jiangsu China; 2 Medical School of Nantong University, 19 Qixiu Road, Nantong, 226001 Jiangsu China

**Keywords:** Hepatocellular carcinoma, Secretory clusterin, β-Catenin, Cancer stem cell, Molecular target

## Abstract

**Background:**

To explore the modulatory effects and mechanism of secretory clusterin (sCLU) on cancer stem cell (CSC) properties in hepatocellular carcinoma (HCC).

**Methods:**

The effects of sCLU repression or overexpression on chemoresistance, migration, invasion, and tumor growth were detected by MTT, wound healing, transwell assays, and xenograft assay, respectively. The tumor sphere assay was performed to evaluate the self-renewal ability of HCC cells. In addition, the molecular regulation between sCLU and AKT/GSK-3β/β-catenin axis in HCC cells were discovered by western blotting, quantitative real-time PCR (qRT-PCR), and immunofluorescence. The expression status of sCLU and β-catenin in HCC tissues were investigated by immunohistochemistry.

**Results:**

Knockdown or overexpressing sCLU remarkably inhibited or promoted the chemoresistance against sorafenib/doxorubicin, metastasis, and tumor growth of HCC cells, respectively. HepG2 and HCCLM3-derived spheroids showed higher expression of sCLU than that in attached cells. Additionally, repressing sCLU impaired the self-renewal capacity of HCC cells and CSC-related chemoresistance while overexpression of sCLU enhanced these CSC properties. Knockdown or overexpression of sCLU inhibited or increased the expressions of β-catenin, cyclinD1, MMP-2 and MMP-9, and the phosphorylation of AKT or GSK3β signaling, respectively. However, LiCl or LY294002 abrogated the effects mediated by sCLU silencing or overexpression on chemoresistance, metastasis, and CSC phenotype. Furthermore, co-expression of sCLU and β-catenin in HCC tissues indicated poor prognosis of HCC patients.

**Conclusions:**

Taken together, the oncogenic sCLU might promote CSC phenotype via activating AKT/GSK3β/β-catenin axis, suggesting that sCLU was a potential molecular-target for HCC therapy.

## Background

Hepatocellular carcinoma (HCC) accounts for 80–90% liver cancers, which ranks the sixth most common neoplasm and the third in terms of cancer-caused death [1]. Patients with cirrhosis, hepatitis B virus (HBV), and hepatitis C virus (HCV) are at the high risk for developing HCC [2]. HCC occurs insidiously at the early stage, remarkably, patients are always diagnosed at the advanced stage with poor prognosis. Sorafenib (an anti-angiogenic and MAP kinase inhibitor) has shown inhibitory effects on a broad spectrum of malignant phenotypes of HCC, including proliferation, angiogenesis, and metastasis [3]. However, low response rate and the high risk of resistance within 6 months limit the efficacy of sorafenib for HCC patients [4]. Therefore, it is critical to identify underlying molecular mechanism of chemotherapy tolerance and novel therapeutic targets against HCC progression.

In past decades, accumulating evidence indicated that cancer stem cells (CSCs) within the tumor bulk were implicated in the recurrence, chemoresistance and metastasis of various cancer types, which is the major hurdles for tumor therapy [5]. Acquisition of stemness properties is involved in HCC progression with loss of differentiated phenotypes [6]. Liver CSCs are enriched in certain defined markers, including epithelial cell adhesion molecule (EpCAM), CD44, CD133, OV6, and Aldehyde Dehydrogenase 1 (ALDH1) [7]. The subpopulations of HCC cells with positive expression of these markers display a spheroid morphology with CSC features, such as high capacity of invasion, self-renewal, tumorigenicity and chemoresistance [8]. Various genes and pathways participate in the maintenance of stem features, which might be considered as potential targets to block CSC-mediated chemoresistance and metastasis of HCC [9].

Recent evidence indicated that secretory clusterin (sCLU) was correlated with CSC properties and chemoresistance in breast cancer cells [10, 11]. sCLU, a heterodimeric sulphated glycoprotein of 70–80 kD, is transcribed from a single-copy gene at 8p21. sCLU is implicated in a variety of biological functions, including lipid transport, senescence, complements cascade, membrane recycling, cell adhesion, and programmed cell death [12, 13]. In particular, sCLU is also linked to HCC processes, due to its roles in anti-apoptosis, angiogenesis and pro-metastasis [14]. In addition, given its excellent performance in diagnosis and prognosis, sCLU has been recommended as a biomarker for HCC [15, 16]. Our previous study showed that sCLU acted as a regulator of AKT/GSK-3β signaling, thereby directing chemoresistance and tumor growth of HCC cells [17]. Hyperactivation of Wnt/β-catenin signaling pathway, the downstream of AKT/GSK-3β, has been frequently associated with CSCs [18]. Interestingly, a possible association between sCLU and Wnt/β-catenin pathway was observed in recent findings: the activity of Wnt/β-catenin pathway was correlated with sCLU expression in prostate cancer cells [19]; moreover, sCLU contributed to multidrug resistance (MDR) of HCC cells via interaction with Wnt/β-catenin pathway [20]. It was proposed that the sCLU might promote the chemoresistance and tumor growth by Wnt/β-catenin-mediated liver CSCs. However, molecular mechanisms in modulation of Wnt/β-catenin and liver CSCs by sCLU remained largely elusive. In the objectives of this study, the effects of sCLU knockdown or overexpression were detected on chemoresistance, metastasis, tumor growth, CSC phenotypes and activity of AKT/GSK-3β/β-catenin axis. In addition, the sCLU and β-catenin expression and prognostic features were investigated in HCC tissues. The study aimed to evaluate sCLU as a CSC “driver” and a potential target for HCC therapy.

## Methods and materials

### Patients and liver specimen

Liver specimens were collected from 72 patients with HCC underwent hepatotomy at the Affiliated Hospital of Nantong University between January 2007 and September 2010. Clinical information was recorded in detail, including each patient’ clinical factors and 5-year post surgery follow-up. The research included 55 men (76.39%) and 17 (23.61%) women with a mean age of 52.3 years (range 31–74 years). The mean diameter of tumors was 4.36 cm (range 0.8–14 cm), and 28 cases (38.89%) were larger than 5 cm. Alpha-fetoprotein (AFP) was elevated (≥ 50 ng/mL) in 36 (50%) patients. Of the patients, there were 22 cases (30.56%) with metastasis and 7 (9.72%) with portal vein invasion; 56 cases (77.78%) with well and moderate differentiation and 16 (22.22%) with poor differentiation; 43 cases (59.72%) at stages I-II and 29 (40.28%) at stages III-IV. HCC was diagnosed histologically in all patients. The study protocol was approved by the ethical committee of Affiliated Hospital of Nantong University. The informed consent was acquired from each participant according to World Medical Association Declaration of Helsinki (World Medical Association, 2013).

### Immunohistochemistry

The tissue microarray (TMA) chip was performed by Outdo Biotech Company (Shanghai, China). 72 cancerous tissues and self-control para-cancerous tissues were involved in the study. After the conformation of tumor area by hematoxylin and eosin (H&E), the target tissues were arrayed into a recipient paraffin block to construct a TMA chip. Immunohistochemistry (IHC) was conducted using Autostainer Universal Staining System (LabVision, USA). TMAs were deparaffinized in xylene, dehydrated in gradient concentrations of ethanol, followed by incubating in 0.3% hydrogen peroxide solution. After that, TMAs were exposed to 10 mM citrate buffer for antigen retrieval, and then blocked in phosphate buffered solution (PBS) containing 10% goat serum at room temperature. Then TMAs were subsequently incubated overnight in solution containing mouse human anti-sCLU monoclonal antibody (1:200, Santa Cruz, USA) or mouse anti-β-catenin monoclonal antibody (1:200, Cell Signaling, USA) at 4 °C. Next, slides were incubated in a horseradish peroxidase-conjugated secondary antibody (1:1000, DAKO, USA) at room temperature for 30 min. At last, TMA sections were visualized by 3,3′-diaminobenzidine (DAB, Kem-En-Tec Diagnostics, Denmark), and counterstained by hematoxylin after dehydration and clearing with xylene. Control samples were treated with PBS instead of primary antibody.

### Evaluation of immunohistochemical results

The IHC results were determined independently by two experienced pathologists under the microscopes (Olympus BX 50, USA), who were blind to each other’s reading. For sCLU, the sum of staining intensity and positive percentages of cells was defined as the final score. In detail, the positive percentages were scored as follows: 0 (0%), 1 for 1% to 33%, 2 for 34% to 66%, and 3 for 67% to 100%; and staining intensity was classified to four categories: 0 for negative, 1 for weak staining, 2 for moderate and 3 for strong staining. Score of 0 to 2 was defined as no or low expression and 3–6 as positive with high expression. The higher score was chosen as the final score in case of a difference between duplicate tissue cores. Meanwhile, it was different for β-catenin: intact membranous expression of β-catenin was defined as negative expression; otherwise, discontinuous membranous staining, cytoplasmic staining, or nuclear staining was regarded as positive expression [21].

### Cell lines and cell culture

HCC cell lines (HCCLM3, MHCC97-H, SMMC7721, and HepG2) were purchased from Type Culture Collection of the Chinese Academy of Sciences (Shanghai, China). Cells were incubated in Dulbecco’s modified eagle medium (DMEM) or Roswell Park Memorial Institute-1640 (RPMI-1640, KeyGen BioTech, Nanjing, China) with solution of penicillin/streptomycin, and 10% fetal bovine serum (FBS) at a 5% CO_2_ incubator with humidified atmosphere.

### Plasmids and transfection

For sCLU silencing, the pRNAT-U6.1/Neo vector was generated previously by Biomics Company (Nantong, China) [17]. The sequences of shRNAs in this study were listed as follows: shRNA-1, 5′-GTAAGTACGTCAATAAGGA-3′; NC-shRNA, 5′-TTCTCCGAACGTGTCACGT-3′. The plasmids of sCLU-overexpressing and control were provided by GenePharma Company (Shanghai, China). Following incubating for 24 h, the cells were transfected with GenJetTM (VerII, SignaGen, USA) according to the manufacturer’s instructions. The transfection efficiency was observed by the fluorescence microscope.

### MTT assay

Cell viability was detected by the MTT assay as described previously [17]. In brief, HepG2 and HCCLM3 (1 × 10^3^/well) were seeded in 96-well plates. The cells were cultured overnight, followed by incubation with sorafenib or doxorubicin at indicated concentrations for 24 h. Then MTT solution was administrated to each well and incubated for 4 h. Optical density (OD) was determined by using a microplate reader at 490 nm.

### Colony formation assay

HepG2 and HCCLM3 cells were plated in 6-well plates with 500 cells per well. The cells were cultured overnight, followed by incubation with sorafenib or doxorubicin and cultured for 12 days. Then the colonies were washed by PBS, followed by fixing in 4% paraformaldehyde, and staining in 0.5% gentian violet. Each experiment was independently conducted at least three times.

### Wound healing assay

Cells were seeded in 6-well plates at the density of 2 × 10^5^ cells/mL. Until confluence, a vertical wound was made by using a sterile pipette tip, followed by washing with medium to remove cell debris. Following the initial picture, the plates were incubated and snapped at indicated time points under a microscope (Olympus, Japan). The width of the wound was measured using a scale bar to detect the relative migration of HCC cells.

### Transwell assay

HepG2 and HCCLM3 cells were collected and resuspended in serum-free medium. Transwell chambers (8 μm, Corning, USA) were pre-treated with Matrigel (BD Biosciences, USA). The lower chamber was added with 600 μL DMEM medium supplemented with 10% FBS, while the upper chambers were plated with HCC cells at the density of 1 × 10^5^ cells/mL. After incubation for 24 h, cells were rinsed in PBS for 3 times, fixed in 4% paraformaldehyde and stained in 0.5% gentian violet. The invasive cells were visualized and counted in 3 random fields under a microscope (Olympus, Japan).

### RNA isolation and qRT-PCR

Total RNA was extracted by using Trizol kit (Invitrogen, USA) according to the manufacturer’s instructions. RNA of each sample was subjected into reverse transcription into synthesized complementary DNA (cDNA) with RevertAidTM cDNA Synthesis Kit (MBI Fermentas, CA). Quantitative real-time PCR (qRT-PCR) was conducted by using SYBR^®^Premix Ex TaqTMII (TaKaRa, Dalian, China) according to the manufacturer’s instructions. Glyceraldehyde-3-phosphate dehydrogenase (GAPDH) was chosen as a reference gene. The melting curves were observed for the non-specific amplification. The messenger RNA (mRNA) expression was determined by using the 2^−ΔΔCt^ method and normalized to respective controls. The primer sequences were as follows. sCLU, Forward (F): 3′-ATCACTGTGACGGTCCCTGTA-5′ and Reverse (R): 3′-TCACTC CTCCCGGTGCTT-5′; CTNNB1, F: 3′-AAGACATCACTGAGCCTGCCAT-5′ and R: 3′-CGATTTGCGGGACAAAGGGCAA-5′; GAPDH, F: 3′-CAAGGTCATCCAT GACAACTTTG-5′ and R: 3′-GTCCACCACCCTGTTGCTGTAG-5′.

### Western blotting

Total protein was extracted by using a radioimmunoprecipitation assay (RIPA) kit (Beyotime, Nanjing, China) with protease inhibitors. Protein samples were loaded in 10% sodium dodecyl sulfate-polyacrylamide gel electrophoresis (SDS-PAGE), followed by transferring onto polyvinylidene difluoride (PVDF) membranes. Then the samples were blocked with 5% bovine serum albumin (BSA) for 3 h, and incubated in the primary antibody overnight at 4 °C. Following washing with Tris-buffered Saline with Tween (TBST) and incubating in the corresponding secondary antibody, the membranes were detected by the enhanced chemiluminescence (ECL) kit (Millipore, USA). Antibodies involved in the study were diluted as follows: GAPDH, β-catenin, GSK-3β, and Cyclin D1 (1:1000; Cell Signaling, USA); sCLU (1:500; Santa Cruz, USA); AKT, p-AKT, p-β-catenin, p-GSK-3β, MMP-2, MMP-9, CD133, CD44, CD90, CD24, and EpCAM (1:1000; Abcam, USA); IgG horseradish peroxidase conjugate (1:1000; Univ-bio, Nanjing, China).

### Tumor sphere formation

HCCLM3 or HepG2 Cells transfected with shRNA-sCLU or OE-sCLU were plated in Ultra-Low Attachment 24-well plate (Conning, USA) and cultured in DMEM/F12 (HyClone, USA) supplemented with 2% B27 (Gibco, USA), N-2(Thermo, USA), 20 ng/mL bFGF/EGF (Sigma, USA). Following the incubation for 2 weeks, the tumor spheres were observed and counted under an inverted microscope (Olympus, Japan).

### Immunofluorescence

HCCLM3 or HepG2 cells were fixed with 4% paraformaldehyde for 30 min at room temperature, followed by blocking in 4% BSA solution, and incubated with primary antibody against β-catenin (dilution ratios: 1: 500) at 4 °C overnight. Then cells were washed and incubated in solution of Alexa Fluor 488 goat anti-rabbit secondary antibody (Keygentec, China). After being washed for 3 times and stained in 4′,6-diamidino-2-phenylindole (DAPI, Sigma, USA) solution for 10 min, the samples were visualized using a fluorescence microscope (Olympus, Japan).

### Xenograft tumor model

The animal study was approved by the Animal Ethics Committee of Affiliated Hospital of Nantong University. Male BALB/c nude mice at the age of 6 weeks were obtained from the animal center of Nantong University. 2 × 10^6^ HepG2 cells transfected with OE-sCLU or control plasmids were suspended in 100 μl PBS and injected subcutaneously into mice. The tumor volume was measured every 4 days and calculated according to the formula: Volume = 0.5 × Length × Width^2^. At the 35th day after injection, the mice were sacrificed. Each resected tumor was analyzed for sCLU and β-catenin protein levels by immunohistochemistry.

### Statistical analysis

The data in this study were presented as mean ± SD and analyzed by the SPSS 19.0 or Stata 12.0 software. Correlations of clinicopathologic variables with sCLU and β-catenin expressions were examined by *χ*^2^ tests. Survival curves were performed by Kaplan–Meier method with a log-rank test. The analyses of univariate and multivariate Cox regression were conducted to evaluate potential prognostic factors. *t* test was evaluated between two groups of continuous variables; the comparisons among three groups were conducted by Student’s Newman-Keuls (SNK) test. *P* value (two-sided tests) less than 0.05 was considered to be statistically significant.

## Results

### Knockdown of sCLU inhibited malignant behaviors of HCC cells

**Fig. 1:**
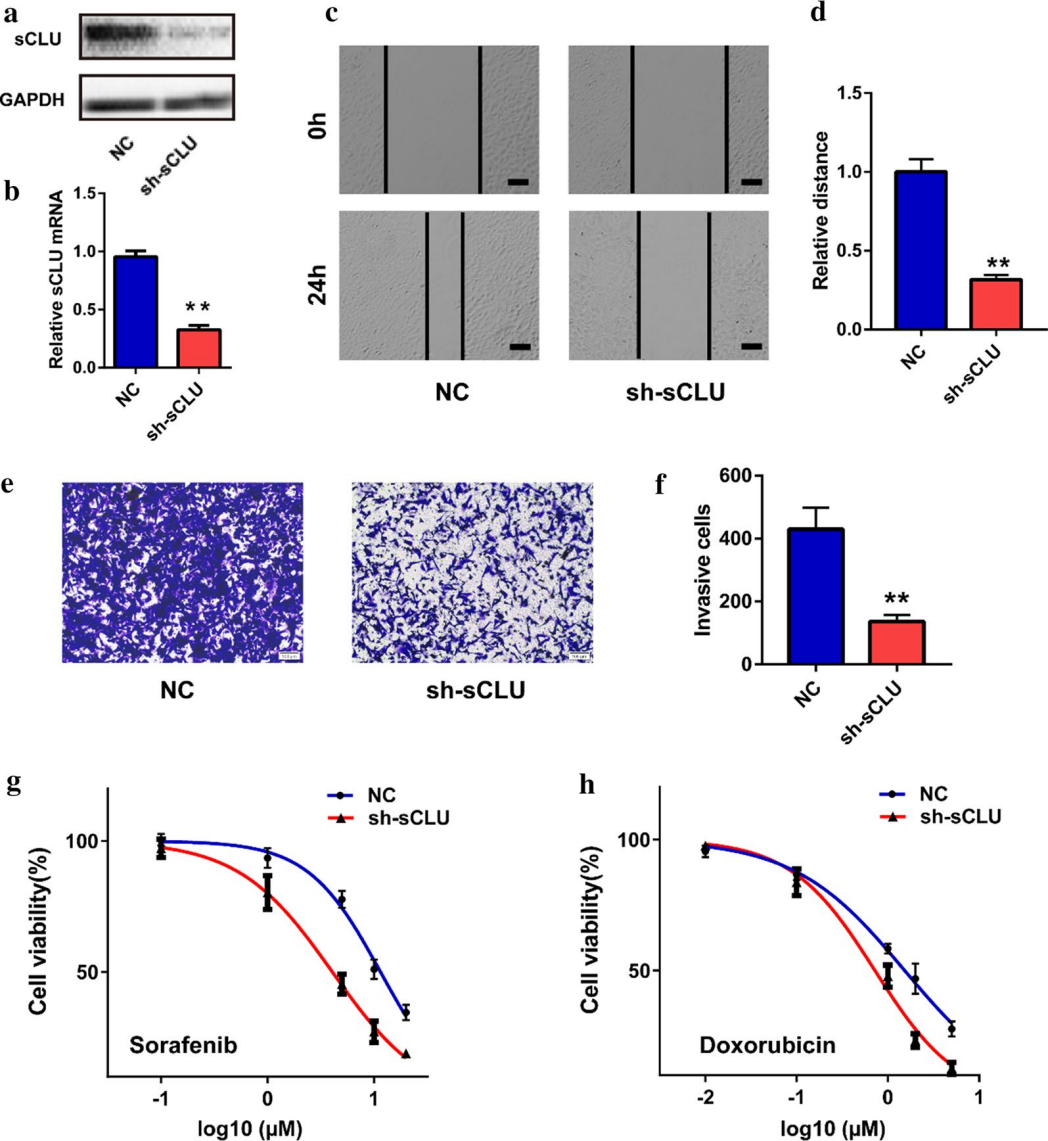
Effects of sCLU knockdown on malignant behaviors of HCC cells. **a** sCLU expression in HCCLM3 cells transfected with NC or sh-sCLU was detected by western blotting. **b** sCLU expression in HCCLM3 cells transfected with NC or sh-sCLU was detected by RT-qPCR. **c** The migration of HCCLM3 cells was detected by wound-healing assay. **d** The relative migration distance in C. **e** The invasion of HCCLM3 cells was detected by transwell assay. **f** The relative invasive cell number in **e**. **g**, **h** MTT assay was used to detect the viability of HCCLM3 cells transfected with NC or sh-sCLU after sorafenib or doxorubicin treatment. Scale bar, 100 µm. **P < 0.01

The effects of silencing sCLU on the biological behaviors of HCC cells are shown in Fig. 1. Our previous work demonstrated that HCCLM3 and HepG2 had relatively higher and lower expression levels of sCLU in HCC cell lines, respectively [17]. Thus, the two cell lines were chosen for further function assays. Transfection with sh-sCLU significantly downregulated the sCLU expression of HCCLM3 cells in protein and mRNA levels (Fig. 1a, b). Further, the wound-healing assay indicated that knockdown of sCLU obviously inhibited the ability of migration of HCCLM3 cells (*t *= 13.92, *P *= 0.0002, Fig. 1c, d). As expected, silencing sCLU also decreased the count of invasive cell according to the transwell assay (*t *= 6.988, *P *= 0.0022, Fig. 1e, f). In addition, the sensitivity of HCCLM3 cells to sorafenib (IC_50_: 11.51 µM vs. 4.062 µM) and doxorubicin (IC_50_: 1.525 µM vs. 0.72 µM) was obviously enhanced following sCLU repression (Fig. 1g, h).

### Overexpression of sCLU enhanced malignant behaviors of HCC cells

**Fig. 2:**
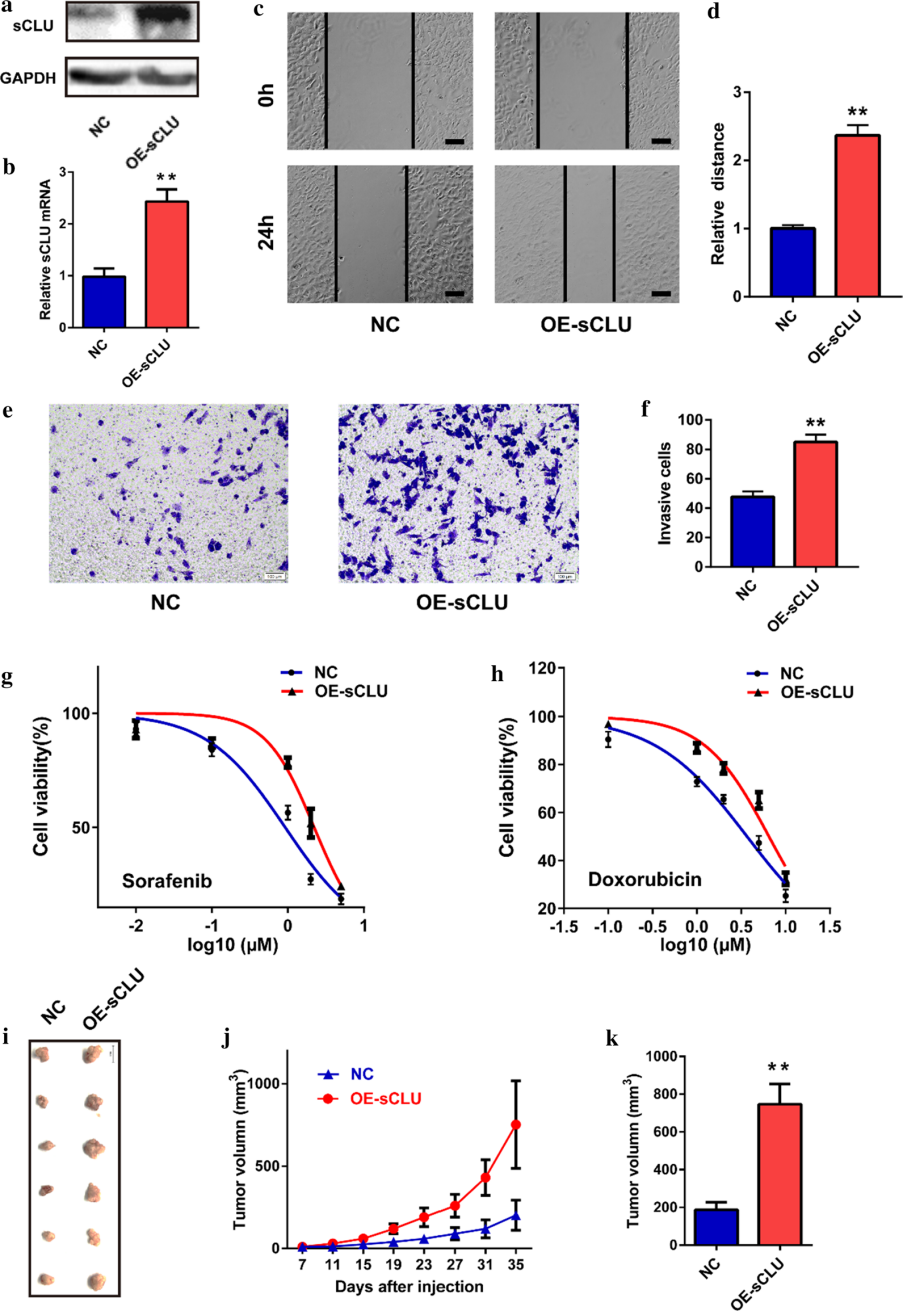
sCLU overexpression contributed to the malignant behaviors of HCC cells. **a** sCLU expression in HepG2 cells transfected with NC or OE-sCLU was detected by western blotting. **b** sCLU overexpression was detected by RT-qPCR. **c** The migration of HepG2 cells transfected with NC or OE-sCLU was detected by wound-healing assay. **d** The relative migration distance in C. **e** The invasion of HepG2 cells was detected by transwell assay. **f** The relative invasive cell number in **e**. **g**, **h** MTT assay was used to detect the viability of HepG2 cells after sorafenib or doxorubicin treatment. **i** The xenograft tumors derived from HepG2 cells transfected with OE-sCLU or NC plasmids. **j** The tumor growth curves of each group identified on the surface of mice. **k** The final volume of each group was calculated. Scale bar, 100 µm. **P < 0.01

The effects of sCLU overexpression on the biological behaviors of HCC cells are presented in Fig. 2. Transfection of the OE-sCLU plasmid remarkably upregulated the sCLU expression of HepG2 cells in protein and mRNA levels (Fig. 2a, b). Following elevating sCLU expression, the migration distance of HepG2 cells was significantly enlarged in the wound-healing assay (*t *= 14.73, *P *= 0.0001, Fig. 2c, d). Moreover, the transwell assay demonstrated that sCLU overexpression obviously promoted the invasion of HepG2 cells (*t *= 10.31, *P *= 0.0005, Fig. 2e, f). As shown in Fig. 2g, h, by contrast, exogenous sCLU caused the chemoresistance of HepG2 cells against sorafenib (IC50: 3.676 µM vs. 6.491 µM) and doxorubicin (IC50: 0.9228 µM vs. 2.218 µM). In addition, OE-sCLU-derived xenograft tumors grew faster than these of the NC group (Fig. 2j). Besides, the mean volume of the final resected tumors in the OE-sCLU group was significantly larger than the NC group (*t *= 4.846, *P *= 0.0007, Fig. 2i, k).

### sCLU was correlated with cancer stem cell properties of HCC

**Fig. 3:**
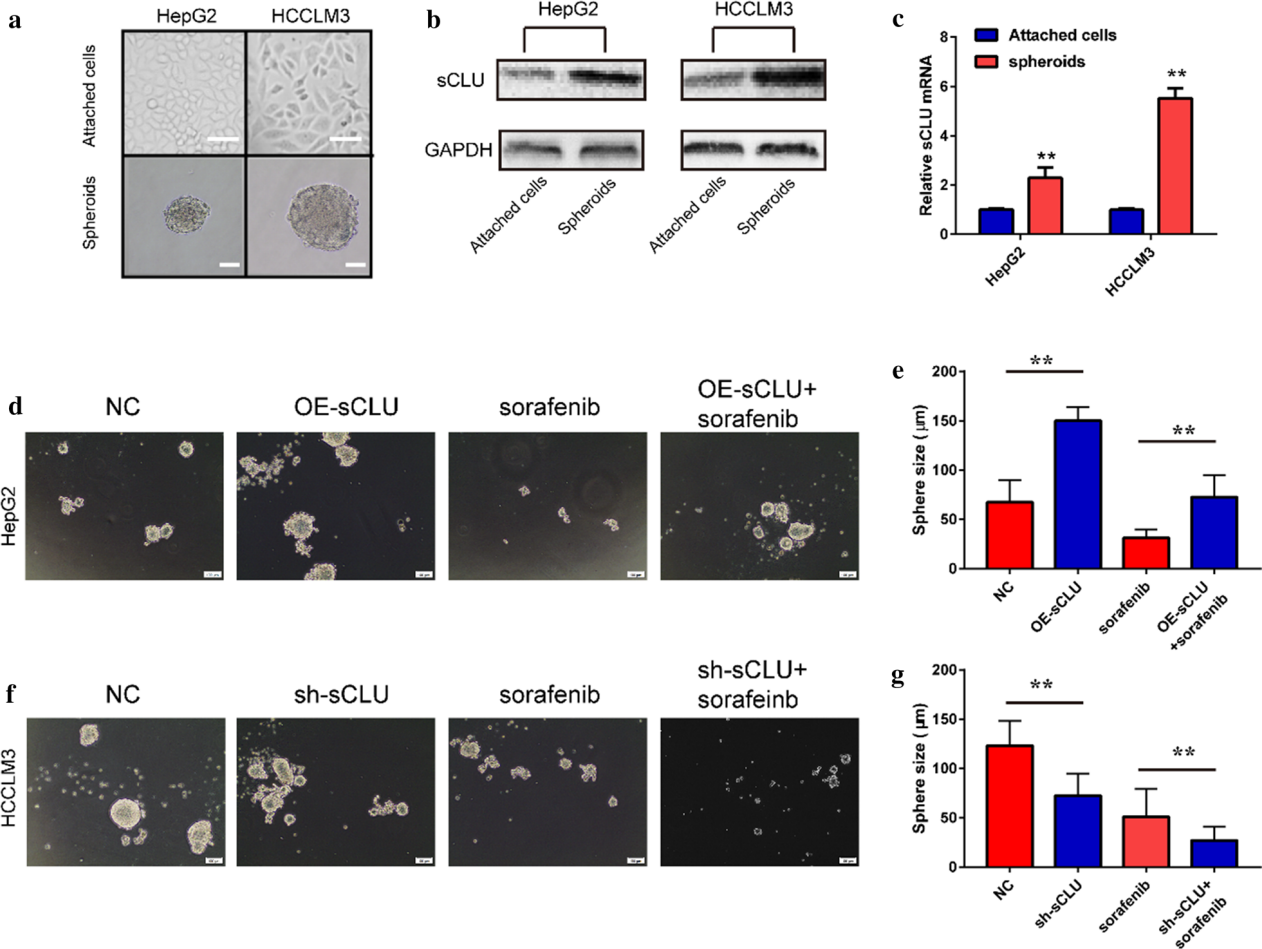
sCLU regulated the cancer stem cell properties of HCC cells. **a** The representative images of tumor spheroids derived from HepG2 and HCCLM3. **b** sCLU expression in attached cells and spheroids was detected by western blotting. **c** sCLU mRNA expression in attached cells and spheroids was detected by RT-qPCR. **d** Tumor spheroids derived from HepG2 cells transfected with NC or OE-sCLU were exposed to sorafenib solution. **e** The mean size of spheroids in **d**. **f** Tumor spheroids derived from HCCLM3 cells transfected with NC or sh-sCLU were exposed to sorafenib solution. **g** The mean size of spheroids in **f**. **P < 0.01

Based on the evidence above, sCLU could promote the metastasis, chemoresistance and tumor growth of HCC cells. Previous studies indicated that sCLU was implicated in the cancer stem cell properties in the breast cancer. The current study explored the association between sCLU and CSC properties of HCC. As shown in Fig. 3a, HepG2 and HCCLM3 cells were used to construct tumor sphere. HCCLM3 cells with higher sCLU expression formed larger spheroids. Western blotting showed that both of HepG2- and HCCLM3- derived tumor spheroids had higher sCLU expression than that of attached HCC cells (Fig. 3b). Elevated sCLU mRNA was also detected in tumor spheroids rather than adherent cells (Fig. 3c). Furthermore, sCLU overexpression enlarged the size of HepG2-derived tumor spheroids with enhanced chemoresistance against sorafenib (Fig. 3d, e). By contrast, knockdown of sCLU decreased the size of HCCLM3-derived tumor spheroids, with upregulating the chemosensitivity against sorafenib (Fig. 3f, g). In addition, inhibiting sCLU significantly downregulated CSC markers of HCC in HCCLM3 cells (CD44, CD133, CD90, EpCAM, and CD24; Additional file 1: Fig. S1A, B). However, overexpression of sCLU up-regulated expression of these markers in HepG2 cells, respectively (Additional file 1: Fig. S1C, D). These evidences indicated that sCLU promoted the CSC properties in HCC.

### sCLU regulated the activity of AKT/GSK-3β/β-catenin axis

**Fig. 4:**
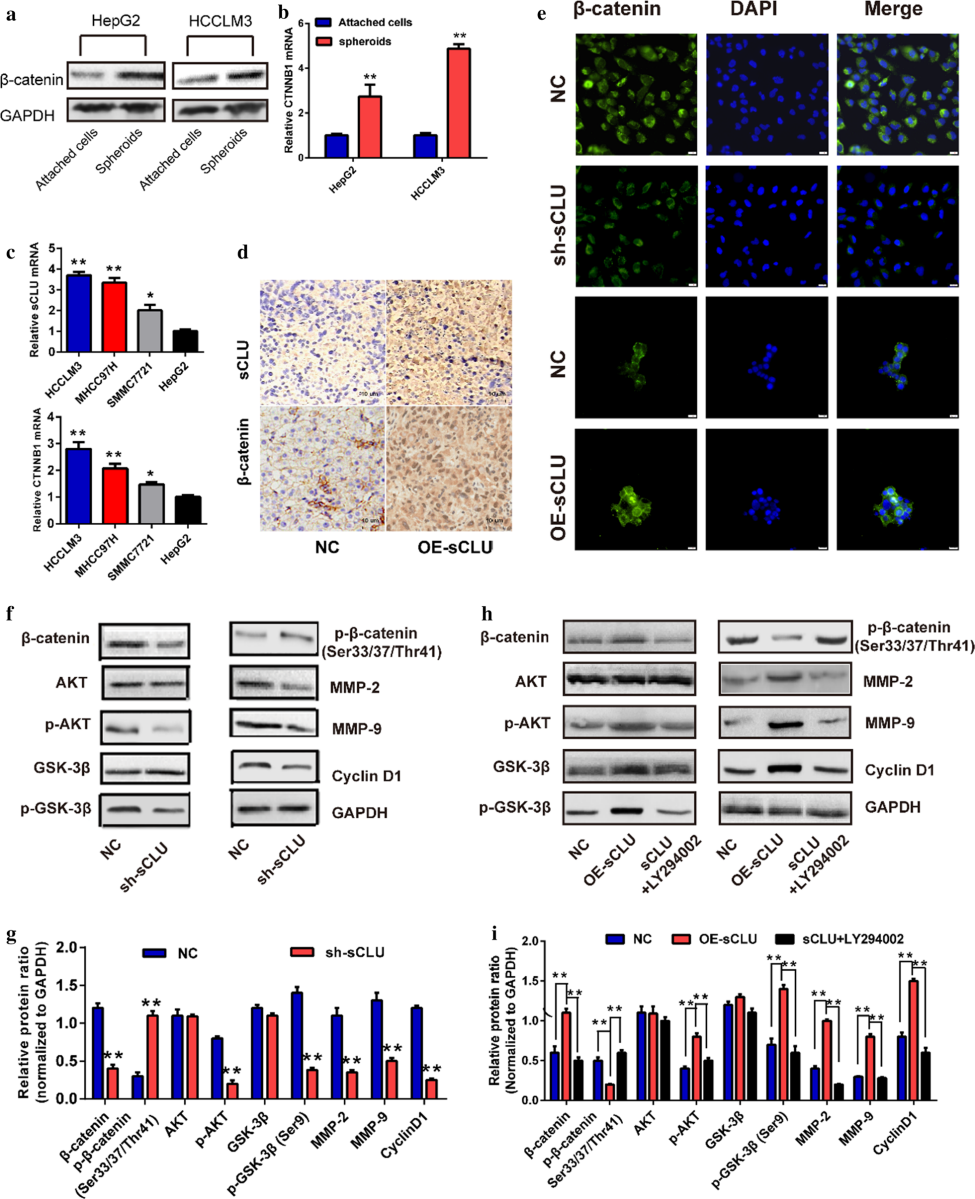
sCLU promoted the activity of β-catenin by AKT/GSK-3β signaling. **a** β-catenin expression in attached cells and spheroids was detected by western blotting. **b** CTNNB1 mRNA expression in attached cells and spheroids was detected by RT-qPCR. **c** sCLU and β-catenin mRNA expressions in HCC cell lines were detected by RT-qPCR. **d** Immunohistochemistry was performed to analyze the expression of sCLU and β-catenin in xenograft tumors. **e** The expressions of β-catenin in sCLU-repressed HCCLM3 cells or sCLU-overexpressed HepG2 cells were determined by immunofluorescence assay. **f** The key proteins of the AKT/GSK-3β/β-catenin axis in sCLU-repressed HCCLM3 cells were measured by western blotting. **g** Relative protein intensities of F were detected by Image J software. **h** The key proteins of the AKT/GSK-3β/β-catenin signaling pathway in sCLU-overexpressed HepG2 cells were measured by western blotting, with or without the treatment of LY294002. **I** Relative protein intensities in H were detected by Image J software. GAPDH was used as a loading control. **P < 0.01

Previous studies indicated that sCLU could regulate the activities of AKT/GSK-3β. However, its downstream β-catenin were essential for the CSC features. Thus, this study further explored the association between sCLU and AKT/GSK-3β/β-catenin axis. Similar with sCLU, HepG2- and HCCLM3- derived tumor spheroids also had higher β-catenin expression than that of attached cells in protein and mRNA level (Fig. 4a, b). In line, sCLU and β-catenin expressions rose consistently with the increase of metastatic potential in HCC cell lines (Fig. 4c). Additionally, IHC of the xenograft tumors above demonstrated that sCLU overexpression also increased β-catenin expression in vivo (Fig. 4d). Based on these observations, sCLU was initially correlated with β-catenin signaling.

The immunofluorescence assay further indicated that knockdown or overexpression of sCLU significantly decreased or increased the β-catenin staining in HCCLM3 and HepG2 cells, respectively (Fig. 4e). Western blotting assay revealed that interference of sCLU repressed β-catenin levels with elevated p-β-catenin levels. Moreover, inhibition of sCLU significantly downregulated the protein levels of p-AKT, p-GSK-3β and downstream genes, including CyclinD1, MMP-2 and MMP-9 (Fig. 4f, g). In contrast, sCLU overexpression significantly increased the expression of β-catenin and its target genes (Fig. 4h, i). Notably, upregulation of sCLU induced the phosphorylation of AKT and GSK-3β, with the downregulation of p-β-catenin. After the treatment of the LY294002, an inhibitor of AKT, abrogated sCLU-induced alterations above. These evidences demonstrated that sCLU could regulate the AKT/GSK-3β/β-catenin axis in HCC cells.

### sCLU promoted CSC properties through AKT/GSK-3β/β-catenin axis

**Fig. 5:**
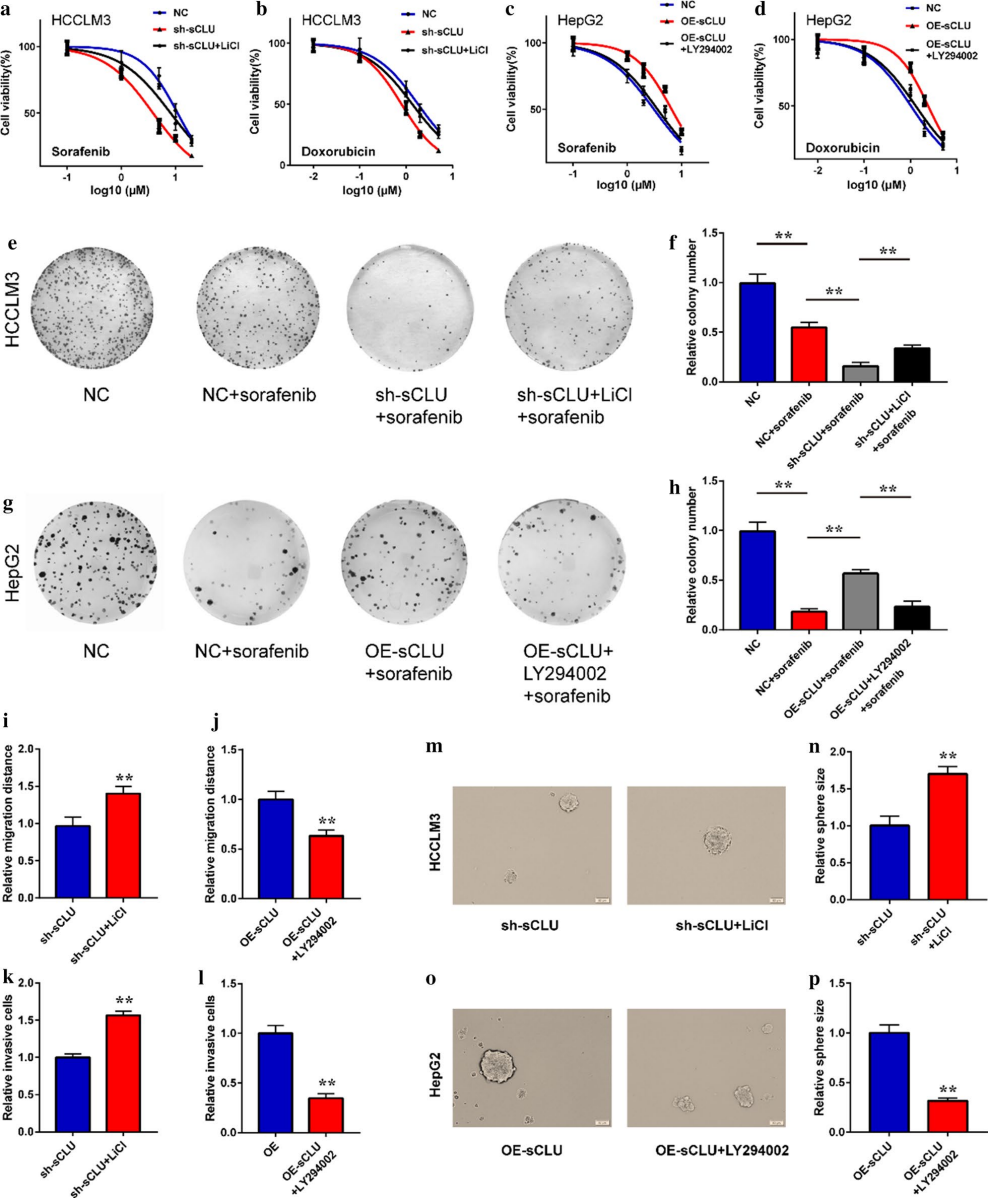
sCLU facilitated cancer stem cell properties of HCC via AKT/GSK-3β/β-catenin Axis. **a**, **b** MTT assay was used to detect the viability of HCCLM3 cells transfected with NC or sh-sCLU after sorafenib or doxorubicin treatment with or without LiCl exposure. **c**, **d** The viability of HepG2 cells transfected with NC or sh-sCLU after sorafenib or doxorubicin treatment with or without LY294002 exposure. **e** The colony formation of HCCLM3 cells after sorafenib or doxorubicin treatment with or without LiCl exposure. **f** The relative colony number in **e**. **g** The colony formation of HepG2 cells after sorafenib or doxorubicin treatment with or without LY294002 exposure. **h** The relative colony number in **g**. **i**, **k** The relative migration and invasion of sCLU-repressed HCCLM3 cells with or without LiCl treatment. **j**, **l** The relative migration and invasion of sCLU-overexpressed HepG2 cells with or without LY294002 treatment. **m** The tumor spheroids derived from sCLU-repressed HCCLM3 cells with or without LiCl treatment. **n** Relative spheroid size in **m**. **o** The tumor spheroids derived from sCLU-overexpressed HepG2 cells with or without LY294002 treatment. **p** Relative spheroid size in **o**. **P < 0.01

Based on the evidences above, our study continued to verify the effects of sCLU- mediated AKT/GSK-3β/β-catenin activation on CSC properties of HCC. As shown in Fig. 5a, b, LiCl, an agonist of GSK-3β/β-catenin, obviously recovered the chemoresistance of HCCLM3 cells, which was impaired by sCLU interference. In contrast, LY294002, an inhibitor of AKT/GSK-3β signaling, abrogated sCLU-induced chemoresistance of HepG2 cells (Fig. 5c, d). Similarly, the colony formation assay demonstrated that silencing sCLU enhanced the efficacy of sorafenib, which was further reversed by LiCl treatment (Fig. 5e, f). However, overexpression of sCLU led to the chemoresistance of HepG2 against sorafenib and abrogated by LY294002 exposure (Fig. 5g, h). In addition, LiCl recovered the inhibitory effects on migration and invasion induced by sCLU knockdown (Fig. 5i, k), while LY294002 inhibited the migration and invasion of sCLU-overexpressed HepG2 cells (Fig. 5j, l). Furthermore, LiCl or LY294002 abrogated the effects of sCLU silencing (Fig. 5m, n) or overexpression (Fig. 5o, p) on CSC tumor sphere formation. Taken together, these results indicated that sCLU might regulate CSC properties via AKT/GSK-3β/β-catenin axis in HCC cells.

### sCLU and β-Catenin expression features in HCC tissues

**Fig. 6:**
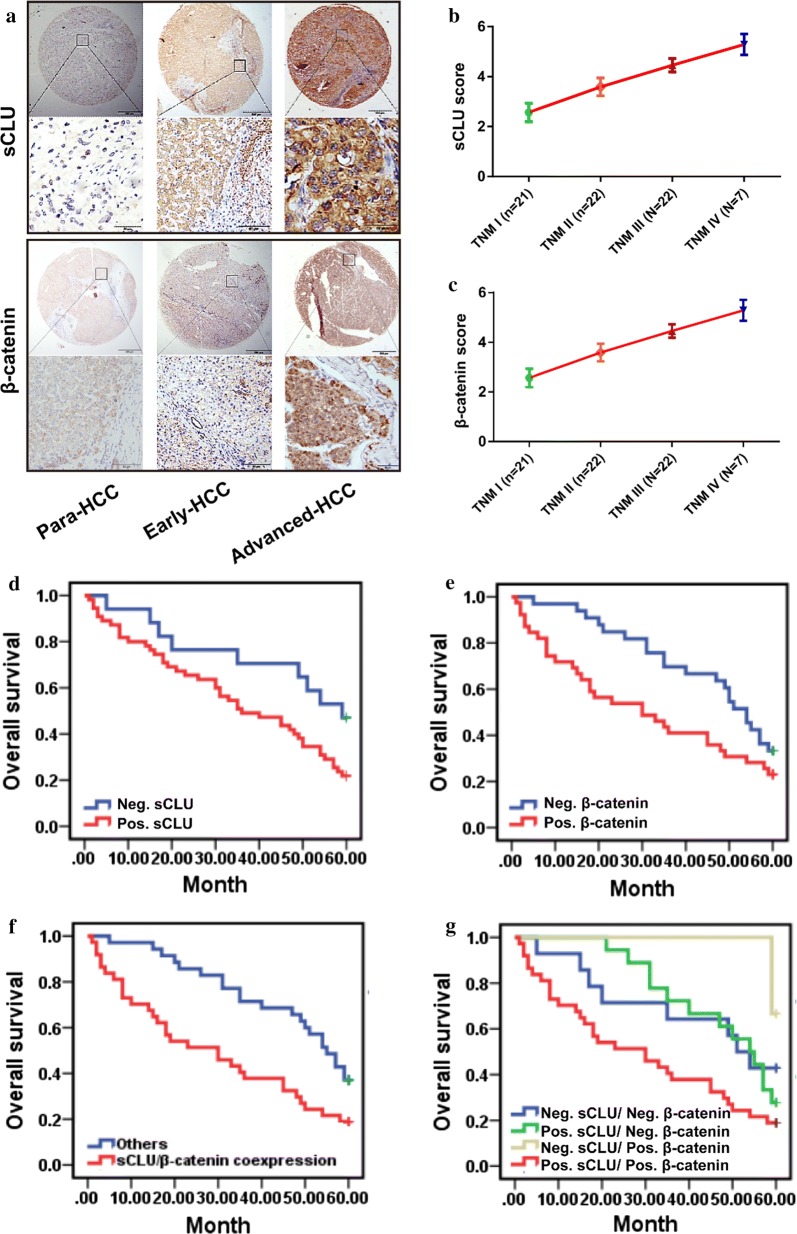
Co-expression of sCLU and β-catenin was correlated with prognosis of HCC patients. **a** Representative immunohistochemical images of sCLU and β-catenin expression in normal tissues and HCC tissues at early or advanced stages. **b** Staining scores of sCLU in HCC tissues with different TNM stages. **c** Staining scores of β-catenin in HCC tissues with different TNM stages. **d** The 5-year overall survival according to sCLU expression (*P *= 0.047). **e** The survival curves according to β-catenin expression (*P *= 0.054). **f** The 5-year overall survival with co-expression of sCLU and β-catenin or other cases (*P *= 0.005). **g** The survival curves of different subgroup based on the expression levels of sCLU and β-catenin (*P *= 0.032). *P < 0.05; **P < 0.01. TNM, tumor-node-metastasis

The sCLU staining was mostly distributed in cell cytoplasm, and HCC tissues with advanced stage had a much stronger brownish yellow staining (Fig. 6a). As demonstrated in Additional file 1: Fig. S2A, C, the positive expression rate of sCLU in para-cancerous tissues was 31.94% (23/72), which was significantly lower than that in HCC tissues (76.39%, 55/72; *χ*^2^= 28.643, *P *< 0.001). Moreover, sCLU expression was elevated in advanced clinical grades of HCC (Fig. 6b). In contrast, normal β-catenin expression was mainly localized in the continuous cell membrane, whereas strong β-catenin staining was observed in cytoplasm and nuclei of tissues at advanced stage. β-catenin also had higher positive expression rate in HCC tissues (55.56%, 40/72) than those in para-cancerous tissues (20.83%, 15/72; *χ*^2^= 18.386, *P *< 0.001; Additional file 1: Fig. S2B, C). Consistent with sCLU, the staining level of β-catenin was also obviously upregulated with HCC staging (Fig. 6c).

Of the 72 HCC patients, 37(51.39%) were positive for simultaneous expressions of sCLU and β-catenin, 18(25.00%) were single-positive for sCLU, 3(4.17%) were single-positive for β-catenin, and 14(19.44%) were negative for either sCLU or β-catenin expression, respectively. Furthermore, the Spearman rank correlation test revealed a positive correlation between the expressions of sCLU and β-catenin in HCC tissues (*P *< 0.001, *r *= 0.424).

### Co-expression of sCLU and β-catenin indicated poor survival of HCC patients

Correlations of sCLU or/and β-catenin expressions with clinicopathologic features in HCC are presented in Table 1. Co-expression of sCLU and β-catenin was significantly associated with lymph node metastasis (*χ*^2^= 15.513, *P *< 0.001), multifocal nodules (*χ*^2^= 4.591, *P *= 0.032), and advanced TNM stage (*χ*^2^ = 19.128, *P *< 0.001). No statistically significant correlation was found with AFP, portal vein invasion, differentiation, HBV infection, tumor size, or liver cirrhosis.

**Table 1:** Correlations of sCLU and β-catenin expressions with clinical factors

Parameters	n	sCLU, n (%)	*P*	β-Catenin, n (%)	*P*	Coexpression, n (%)	*P*
AFP(µg/L)			0.781		0.343		0.238
< 50	36	28 (77.78)		18 (50.00)		16 (44.44)	
≥ 50	36	27 (75.00)		22 (61.11)		21 (58.33)	
Portal vein invasion			0.745		0.792		0.749
With	7	5 (71.43)		4 (57.14)		4 (57.14)	
Without	65	50 (76.92)		36 (55.38)		33 (50.77)	
HBsAg			0.429		0.482		0.768
Positive	28	20 (71.43)		17 (60.71)		15 (53.57)	
Negative	44	35 (79.55)		23 (52.27)		22 (50.00)	
Tumor size (cm)			0.137		0.570		0.207
< 5	44	31 (70.45)		23 (52.27)		20 (45.45)	
≥ 5	28	24 (85.71)		17 (60.71)		17 (60.71)	
Liver cirrhosis			0.262		0.273		0.220
With	54	43 (79.63)		32 (59.26)		30 (55.56)	
Without	18	12 (66.67)		8 (44.44)		7 (38.89)	
Metastasis			*0.012*		*< 0.001*		*< 0.001*
With	22	21 (95.45)		19 (86.36)		19 (86.36)	
Without	50	34 (68.00)		21 (42.00)		18 (36.00)	
Differentiation			0.235		0.228		0.115
Poor	16	14 (87.50)		11 (68.75)		11 (68.75)	
Others	56	41 (73.21)		29 (51.78)		26 (46.43)	
Multifocal			0.235	5.500	*0.019*		*0.032*
Yes	16	14 (87.50)		13 (81.25)		12 (75.00)	
No	56	41 (73.21)		27 (48.21)		25 (44.64)	
TNM stage			*< 0.001*		*0.001*		*< 0.001*
I and II	43	26 (60.47)		17 (39.53)		13 (30.23)	
III and IV	29	29 (100.00)		23 (79.31)		24 (82.76)	

*AFP* α-fetoprotein, *TNM* tumor-node-metastasis, *sCLU* secretory clusterin

Italics, *P *< 0.05

As shown in Fig. 6d, the sCLU-positive HCC patients had shorter survival than negative ones (*χ*^2^= 4.433, *P *= 0.047). On the other hand, β-catenin-positive patients had a poorer OS, although the difference was not significant (Fig. 6e, *χ*^2^= 3.425, *P *= 0.054). Moreover, co-expression of sCLU and β-catenin revealed an obviously shorter overall survival (Fig. 6f, *χ*^2^= 3.425, *P *= 0.005). Furthermore, patients were split into 4 groups according to the expressions of the sCLU and β-catenin: sCLU (+)/β-catenin (+), sCLU (+)/β-catenin (−), sCLU (−)/β-catenin (+), and sCLU (−)/β-catenin (−); the Kaplan–Meier assay showed that patients with sCLU (+)/β-catenin (+) had the poorest OS among the groups (Fig. 6g, *χ*^2^= 11.469, *P *= 0.032).

The univariate and multivariate Cox Regression analyses are presented in Table 2. The univariate analysis indicated that co-expression of sCLU and β-catenin, vascular invasion, lymph node metastasis, multifocal nodules and TNM stage were potentially risk factors for HCC. Next, multivariate Cox regression analysis showed that sCLU/β-catenin co-expression was correlated with poor survival and could serve as an independent biomarker for the subset of HCC patients (hazard ratio, 1.965; 95% CI 1.050–3.678; *P *= 0.035).

**Table 2:** Univariate and multivariate survival analysis

	Univariate analysis			Multivariable analysis (adjusted for age and sex)		
	HR	*P *> |Z|	95% CI	HR	*P *> |Z|	95% CI
AFP (ng/mL)						
<50 vs. ≥ 50	1.613	0.088	0.932–2.792	–	–	–
Tumor size						
< 5 cm vs. ≥ 5 cm	1.711	0.056	0.987–2.968	–	–	–
Differentiation						
Poor vs. others	1.011	0.974	0.530–1.928	–	–	–
Liver cirrhosis						
With vs. without	0.998	0.994	0.532–1.869	–	–	–
Portal vein invasion						
With vs. without	4.063	*0.001*	1.768–9.339	2.393	0.091	0.869–6.590
Lymph node metastasis						
With vs. without	3.438	*< 0.001*	1.933–6.117	1.677	0.347	0.571–4.922
HBsAg						
With vs. without	0.856	0.949	0.539–1.672	–	–	–
TNM						
I and II vs. III and IV	3.056	*< 0.001*	1.756–5.321	1.474	0.407	0.589–3.690
Multifocal						
Yes vs. without	2.815	*0.001*	1.542–5.140	1.235	0.640	0.510–2.989
sCLU expression						
Positive vs. negative	2.030	0.054	0.987–4.175	–	–	–
β-catenin expression						
Positive vs. negative	1.700	0.060	0.979–2.953	–	–	–
sCLU/β-catenin co-expression						
Positive vs. negative	2.155	*0.007*	1.237–3.752	1.965	*0.035*	1.050–3.678

*CI* confidence interval, *HR* hazard ratio, *AFP* α-fetoprotein, *TNM* tumor-node-metastasis, *sCLU* secretory clusterin

P-values in italic indicate statically significant

## Discussion

Carcinogenesis of HCC is a complicated, multi-factorial, and multi-staged process, and oncogenes always contribute to tumor progression through varieties of cancer-related pathways [22]. sCLU, a stress-activated molecular chaperone, is known with its cell-aggregating activity when first separated from ram rete testis fluid [23]. However, recent studies indicated that sCLU was overexpressed in the aggressive neoplasm and played an important part in cancer progression and metastasis [24, 25]. Consistently, the current study demonstrated that knockdown or overexpressing sCLU inhibited or promoted the chemoresistance against sorafenib/doxorubicin, metastasis, and tumor growth, respectively. Given sCLU-mediated malignant behaviors and the correlation of sCLU with CSC in other cancer type, our study further explored the relationship between sCLU and liver CSCs. As expected, HCC cells-derived spheroids showed higher sCLU expression than that in attached cells. Additionally, repressing sCLU inhibited the self-renewal capacity of HCC cells while overexpression of sCLU promoted the CSC properties. Interestingly, sCLU overexpression also protected HCC cells-derived spheroids from sorafenib. These evidences were consistent with previous studies, revealing that sCLU facilitated the CSC-mediated phenotypes.

Various pathways are implicated in the CSC features, of which hyperactivated Wnt/β-catenin pathway plays indispensable roles. Wnt/β-catenin transduction pathway is considered to regulate cell morphology and differentiation in normal condition [26, 27]. However, when it comes to hyperactivation, aberrant β-catenin accumulated in the cytoplasm will be transported into nuclear binding to the LEF/TCF nuclear transcription factor family members. It subsequently stimulates the downstream oncogenes to induce more aggressive phenotypes, including proliferation, metastasis and CSC phenotypes [28]. Our previous work and other studies demonstrated that sCLU could activate AKT and subsequently inactive GSK3β via phosphorylation in ser9 [17, 29]. It has been investigated that GSK3β, a join-point between PI3K/AKT pathway and Wnt/β-catenin pathway, is essential for the stability of β-catenin in cytoplasm [30, 31]. sCLU has been associated to the activity of Wnt pathway and β-catenin expression by previous studies [19]. Based on it, we hypothesized that sCLU might regulate CSC phenotype via AKT/GSK3β/β-catenin axis. As expected, sCLU was observed to activate PI3K/AKT signaling pathway and lead to the phosphorylation (inactivation) of GSK3β, resulting in the stabilization of β-catenin protein and upregulation of downstream genes. sCLU overexpression also led to the upregulation of β-catenin in the xenograft tissues. In addition, an inhibitor of AKT LY294002 reversed the boost of β-catenin and its transcript activity induced by sCLU. Furthermore, LiCl (GSK-3β agonist) or LY294002 abrogated the effects induced by sCLU silencing or overexpression on chemoresistance, metastasis and self-renewal ability of CSCs. These evidences indicated that sCLU might induce CSC phenotype through AKT/GSK3β/β-catenin axis.

**Fig. 7:**
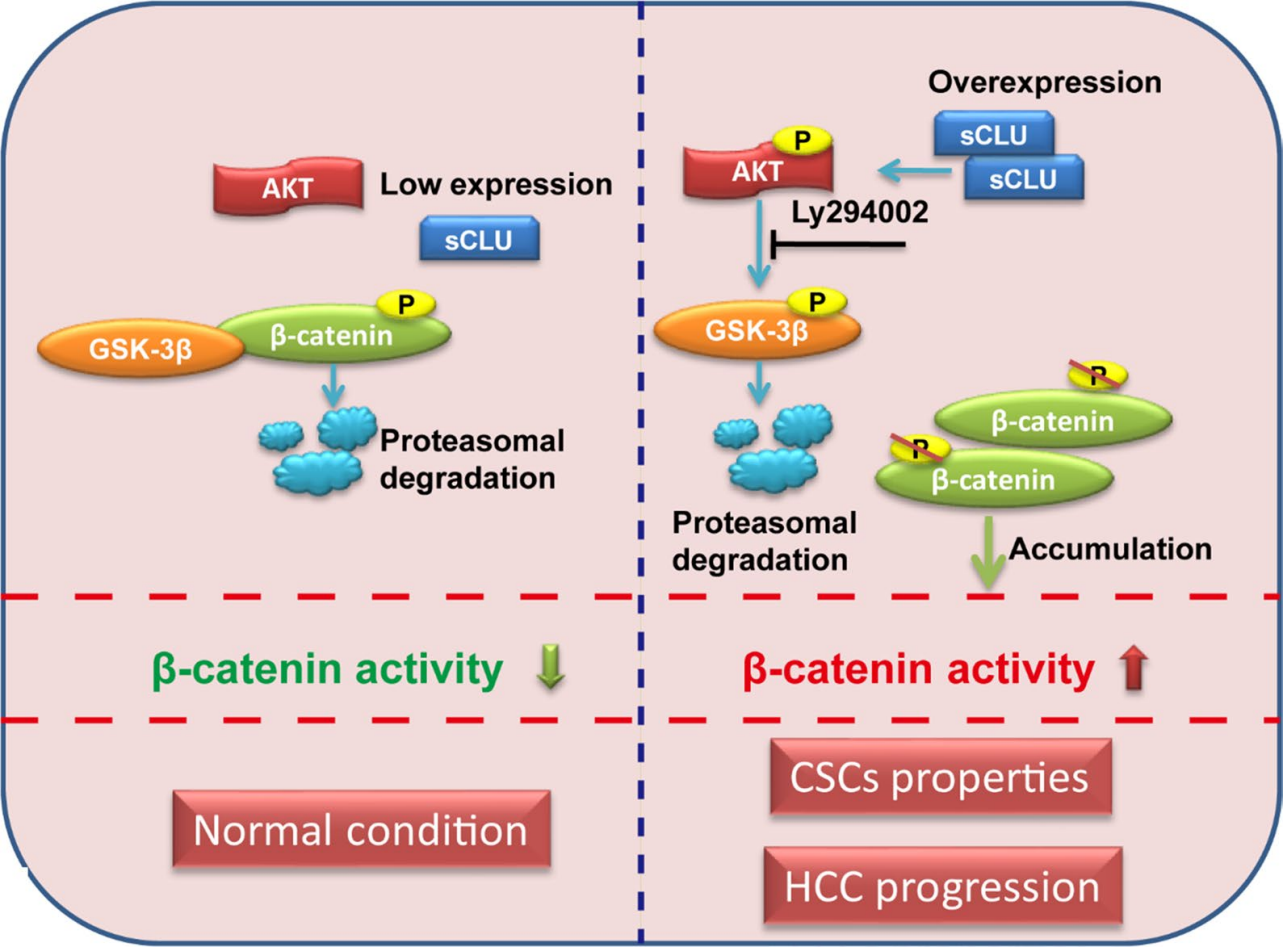
Hypothetical model illustrates the effects of sCLU on AKT/GSK-3β/β-catenin Axis. In hepatocarcinogenesis, sCLU overexpression in cytoplasm leads to the phosphorylation of AKT. Subsequently, activated AKT further phosphorylates GSK-3β, which prevents β-catenin from GSK-3β-mediated phosphorylation and degradation. The accumulation of β-catenin enhances Wnt/β-catenin-mediated CSC properties of HCC cells, results in chemoresistance and metastasis, and eventually promotes the progression of HCC

Based on these results above, sCLU-mediated AKT/GSK3β/β-catenin activation contributed to the malignant behaviors and CSC phenotype of HCC cells. Then we further explored expression characteristics of sCLU and the key factor of Wnt pathway in HCC tissues. sCLU and β-catenin had a parallelly upregulated expression trend in tissues at advanced stages than in para-cancerous tissues or early stages, indicating that both of them were involved in HCC staging. However, despite that the prognostic value of the two proteins was previously reported, the current analysis found that neither of sCLU nor β-catenin alone was an independent prognostic marker. We speculated that the discrepancy might be attributed to the sample size and differences in evaluation of IHC. Interestingly, the subset of HCC patients with simultaneous expression had poorest prognosis compared with other patients, further indicating sCLU and β-catenin contributed to the progression of HCC. Given above, it was speculated that there might be a positive correlation between sCLU and AKT/GSK-3β/β-catenin pathway during HCC progression, which could be activated to cause CSC phenotype and other malignant behaviors (Fig. 7).

## Conclusions

In conclusion, our study demonstrated for the first time that sCLU activated AKT/GSK3β/β-catenin axis, followed by inducing chemoresistance, metastasis, CSC phenotype, and even poor prognosis of HCC patients. While more assays should be conducted to validate the exact mechanisms, the study sheds lights on the novel approach to block liver CSC phenotype and an attractive strategy for HCC therapy.

## Supplementary information

**Additional file 1: Fig. S1.** sCLU regulated the expression of CSC markers of HCC cells. A, the expression of CSC markers in HCCLM3 cells transfected with NC or sh-sCLU were detected by western blotting. B, relative protein intensities in A were detected by Image J software. C, the expression of CSC markers in HepG2 cells transfected with NC or OE-sCLU were detected by western blotting. D, relative protein intensities in C were detected by Image J software. GAPDH was used as a loading control. **P < 0.01; *P < 0.05. **Fig. S2.** The expression features of sCLU and β-catenin in HCC tissues. **A,** sCLU staining scores in HCC and para-cancerous tissues. **B,** β-catenin staining scores in HCC and para-cancerous tissues. **C,** positive ratio of sCLU and β-catenin expression in HCC tissues at different TNM stages. *P < 0.05, **P < 0.01. TNM, tumor-node-metastasis.

## Data Availability

The datasets generated for this study are available on request to the corresponding author.

## Abbreviations

sCLU: Secretory clusterin
HCC: Hepatocellular carcinoma
CSC: Cancer stem cell
HBV: Hepatitis B virus
EpCAM: Epithelial cell adhesion molecule
ALDH1: Aldehyde Dehydrogenase 1
TMA: Tissue microarray
IHC: Immunohistochemistry
PBS: Phosphate buffered solution
DMEM: Dulbecco’s modified eagle medium
FBS: Fetal bovine serum
mRNA: The messenger RNA
PVDF: Polyvinylidene difluoride
SNK: Student’s Newman-Keuls
