# Supplementary figures and images for: Secretory clusterin promotes hepatocellular carcinoma progression by facilitating cancer stem cell properties via AKT/GSK-3β/β-catenin axis
Source: J Transl Med. 2020 Feb 14;18:81. doi: 10.1186/s12967-020-02262-7 (PMC7023808; doi:10.1186/s12967-020-02262-7)

**Figure S1. Zheng et al.**


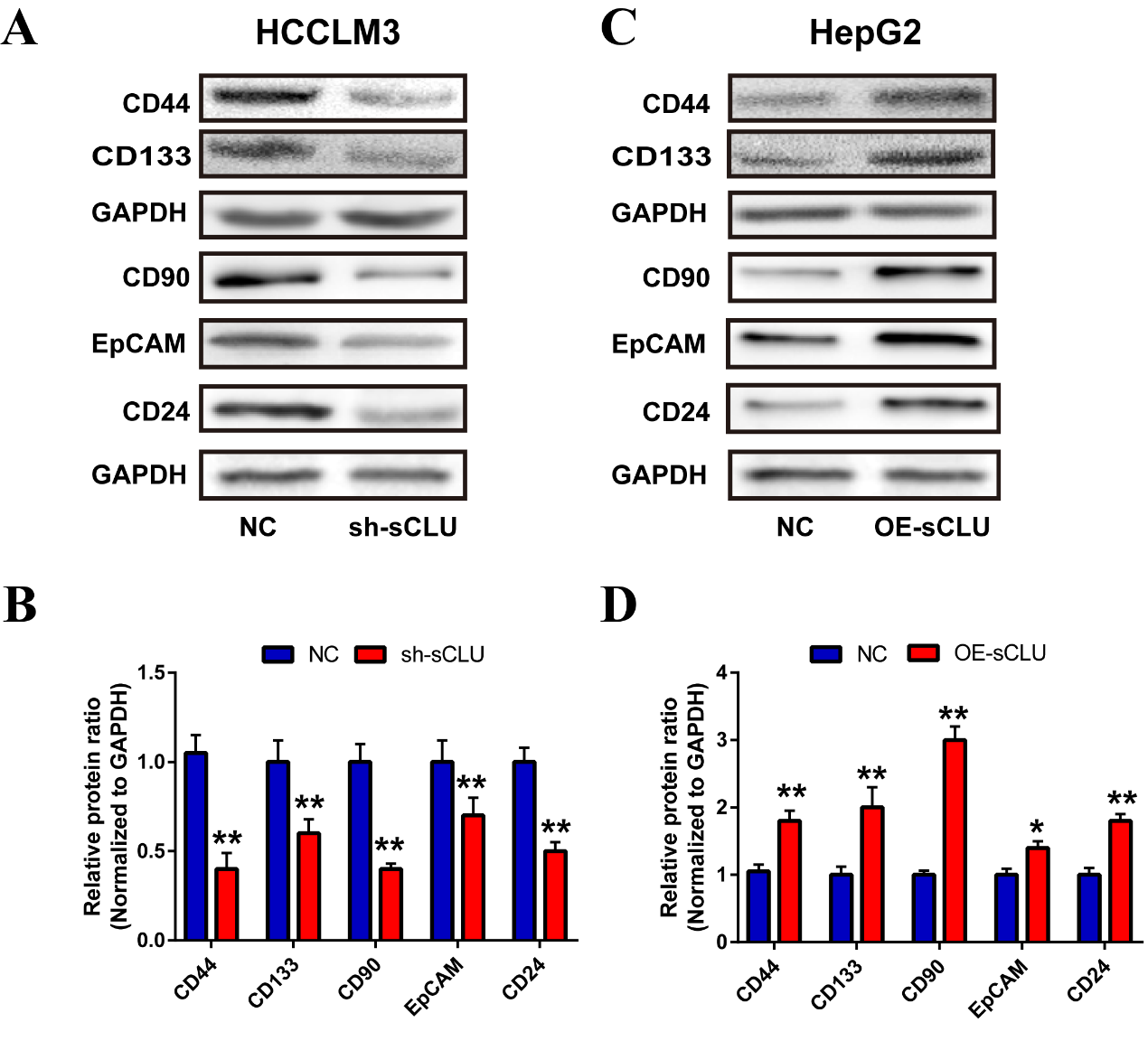


**Figure S2. Zheng et al.**

**
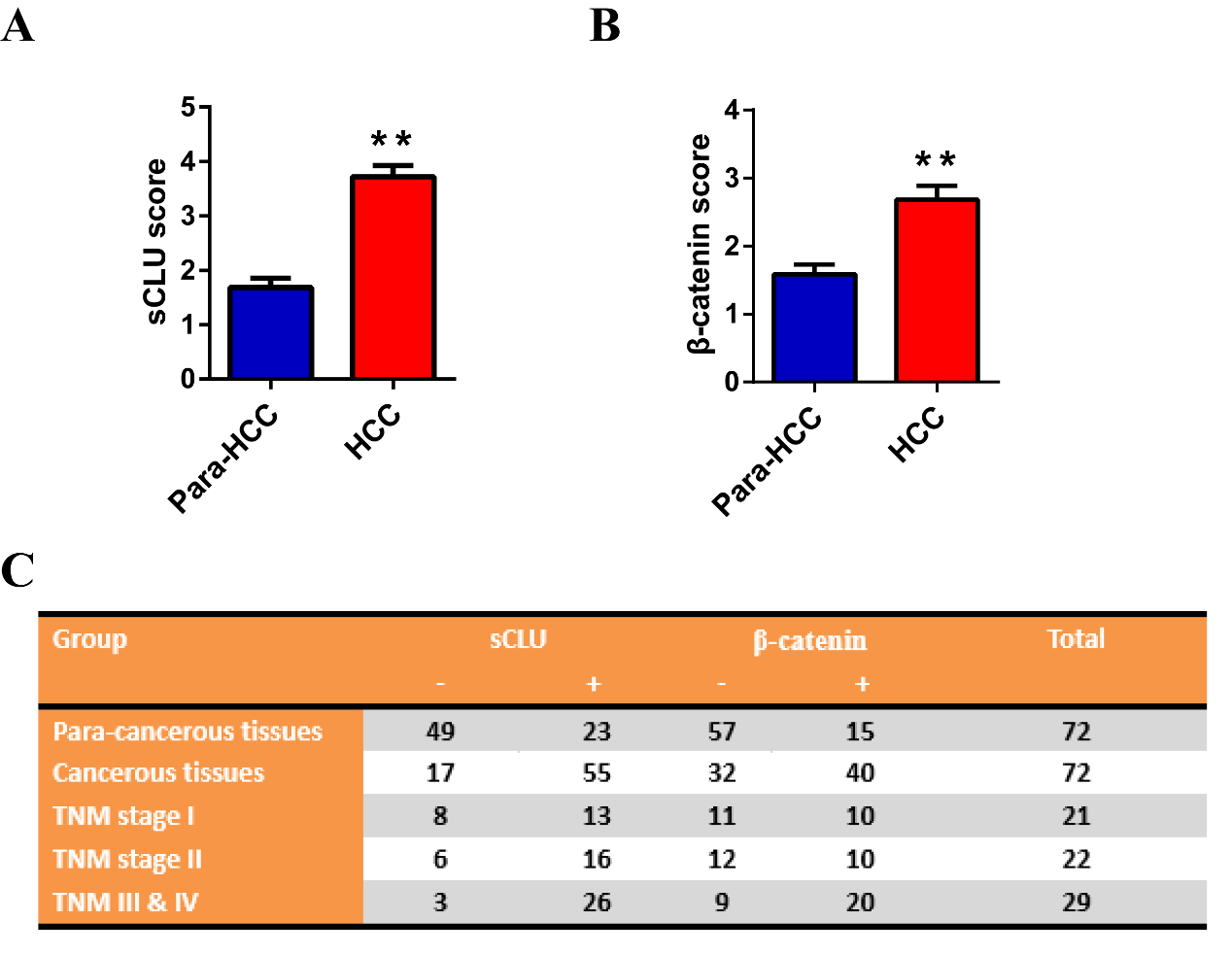
**

Supplement: Supplementary file 1 — Additional file 1: Fig. S1. sCLU regulated the expression of CSC markers of HCC cells. A, the expression of CSC markers in HCCLM3 cells transfected with NC or sh-sCLU were detected by western blotting. B, relative protein intensities in A were detected by Image J software. C, the expression of CSC markers in HepG2 cells transfected with NC or OE-sCLU were detected by western blotting. D, relative protein intensities in C were detected by Image J software. GAPDH was used as a loading control. **P < 0.01; *P < 0.05. Fig. S2. The expression features of sCLU and β-catenin in HCC tissues. A, sCLU staining scores in HCC and para-cancerous tissues. B, β-catenin staining scores in HCC and para-cancerous tissues. C, positive ratio of sCLU and β-catenin expression in HCC tissues at different TNM stages. *P < 0.05, **P < 0.01. TNM, tumor-node-metastasis. [file 12967_2020_2262_MOESM1_ESM.docx]
